# Bupivacaine in combination with sildenafil (Viagra) and vitamin D3 have anti-inflammatory effects in osteoarthritic chondrocytes

**DOI:** 10.1016/j.crphar.2021.100066

**Published:** 2021-10-19

**Authors:** Elisabeth Hansson, Eva Skiöldebrand

**Affiliations:** a Department of Clinical Neuroscience, Institute of Neuroscience and Physiology, The Sahlgrenska Academy, University of Gothenburg, Gothenburg, Sweden; b Department of Pathology, Institute of Biomedical Sciences and Veterinary Public Health, Swedish University of Agricultural Sciences, Uppsala, Sweden; c Department of Clinical Chemistry and Transfusion Medicine, Institute of Biomedicine, Sahlgrenska University Hospital, Gothenburg University, Gothenburg, Sweden

**Keywords:** Drug treatment, Chondrocytes, Inflammation, Ca^2+^ signaling, TLR4

## Abstract

**Aims:**

To treat osteoarthritic chondrocytes and thereby reduce the inflammation with a drug combination that primarily affects 5-HT- and ATP-evoked Ca^2+^ signaling. In osteoarthritic chondrocytes, Ca^2+^ signaling is elevated, resulting in increased production of ATP and inflammatory mediators. The expression of TLR4 and Na^+^/K^+^-ATPase was used to evaluate the inflammatory status of the cells.

**Main methods:**

Equine chondrocytes were collected from joints with mild structural osteoarthritic changes and cultured in monolayers. The cells were treated with a combination of bupivacaine (1 pM) and sildenafil (1 ​μM) in combination with vitamin D3 (100 ​nM). A high-throughput screening system, the Flexstation 3 microplate reader, was used to measure intra- and extracellular Ca^2+^ signaling after exposure to 5-HT, glutamate, or ATP. Expression of inflammatory receptors was assessed by Western blotting.

**Key findings:**

Drug treatment substantially reduced 5-HT- and ATP-evoked intracellular Ca^2+^ release and TLR4 expression compared to those in untreated chondrocytes. The combination of sildenafil, vitamin D3 together with metformin, as the ability to take up glucose is limited, increased Na^+^/K^+^-ATPase expression.

**Significance:**

The combination of these three therapeutic substances at concentrations much lower than usually used, reduced expression of the inflammatory receptor TLR4 and increased the cell membrane enzyme Na^+^/K^+^-ATPase, which regulates cell volume and reduces increased intracellular Ca^2+^ concentrations. These remarkable results indicate that this drug combination has disease-modifying osteoarthritis drug (DMOAD) properties and may be a new clinical therapy for osteoarthritis (OA).

## Highlights

• Aim: reduce inflammation in osteoarthritic chondrocytes with a low-dose drug combination.
• Bupivacaine, sildenafil, vitamin D3, and metformin showed anti-inflammatory effects.
• The drug combination has disease-modifying osteoarthritis drug (DMOAD) properties.
• This drug combination may be a new clinical therapy for osteoarthritis (OA).

## Graphical abstract

Schematic illustration highlighting inflammatory OA chondrocytes (A). Intracellular Ca^2+^ release is increased, resulting in Ca^2+^ oscillations. As a result, the production and release of ATP and proinflammatory cytokines increase. This in turn leads to extracellular Ca^2+^ signaling by the stimulation of ATP receptors. Intercellular Ca^2+^ signaling through gap junctions is decreased (Mobasheri et al., 2002). Glucose uptake into chondrocytes is decreased (Nakamura, 2002). (B) The local anesthetic agent bupivacaine (1 pM) reduces Ca^2+^ oscillations after stimulation with 5-HT. Intercellular Ca^2+^ signaling increases through gap junctions, and glucose uptake is increased. (C) Effects of sildenafil (1 ​μM), which blocks PDE-5 and increases the production of cGMP. This in turn increases protein kinase G (PKG) and stimulates the Na^+^/K^+^-ATPase pump. The expression of TLR4 is decreased. Even here the glucose uptake is increased. Through some mechanism, ATP production decreases (Hansson et al., 2018).

**Figure:**
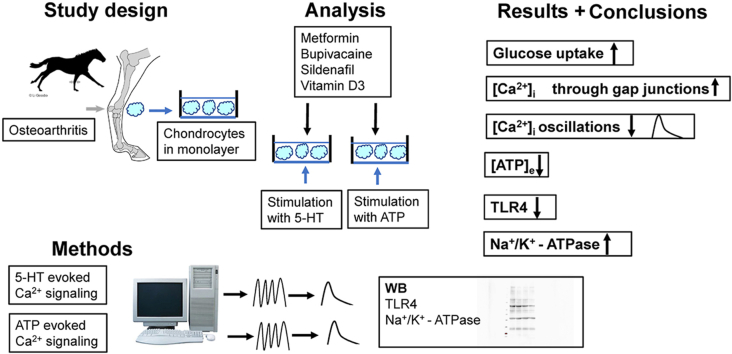

## 1 Introduction

Osteoarthritis (OA) is a chronic progressive disease that leads to severe joint pain and the loss of joint mobility in horses and humans (Goldring and Goldring, 2007; van Weeren and de Grauw, 2010). So far, no disease-modifying OA drugs (DMOADs) are available (MacKay et al., 2018). The multifactorial etiology includes biomechanics, overload to the joint, low-grade systemic inflammation and activation of the body's immune system. Toll-like receptors (TLRs), particularly TLR4, have been identified as potential drug targets for the treatment of inflammatory diseases such as OA (ul Ain et al., 2020). We previously showed that chondrocytes increase their intracellular Ca^2+^ release, which is associated with increased expression of TLR4 and inflammatory mediators (Skiöldebrand et al., 2018). The balance between cell-to-cell Ca^2+^ signaling and extracellular Ca^2+^ signaling due to increased ATP production and release is changed (Skiöldebrand et al., 2019; Cotrina et al., 2000; Beamer et al., 2017).

Chondrocytes are responsible for the synthesis, assembly and turnover of extracellular matrix (ECM) (Roberts et al., 2019) and production of proinflammatory cytokines (Goldring and Otero, 2011; Wodell-May and Sommerfeld, 2020). Damage-associated molecular patterns (DAMPs) are produced locally in the joint and signal through TLRs, thereby mediating and sustaining the inflammatory response (Kielian, 2006). An excessive breakdown of ECM results in fragments, which act as signaling molecules (Miller et al., 2019).

Changes in intracellular Ca^2+^ influence matrix synthesis and promote the release of signaling molecules, such as neurotransmitters and cytokines (Zhou et al., 2018; Patetsos and Horjales-Araujo, 2016). Na^+^/K^+^-ATPase functions as an energy-requiring ionic pump that actively transports cations across the cell membrane and regulates cell volume. In inflammation, this pump is downregulated, and the resultant influx in water results in cell edema (Zachary, 2017).

The horse is an excellent research animal for studying OA since the horse spontaneously develops this disease due to biomechanical overload to the joint, and the pathogenesis of the disease is equal in humans and horses. The horse genome was fully sequenced in 2009, revealing a genome structure with similarities to that of humans (Wade et al., 2009). The horse has been approved by the Food and Drug Administration (FDA) as an animal model to study disease mechanisms in humans. The possibility of harvesting cartilage specimens and chondrocytes from articular cartilage from euthanized horses within 24 ​h postmortem gives us vast access to material with early stages of OA.

This study aimed to treat equine OA chondrocytes with or without lipopolysaccharide (LPS) stimulation, an endotoxin that can be used to experimentally generate an inflammatory response (Nakamura, 2002) that stimulates TLR4 (Skiöldebrand et al., 2018). A drug combination of bupivacaine and sildenafil, both in low concentrations, are used. The drug combination primarily affected 5-HT- and ATP-evoked intracellular Ca^2+^. However, it also influenced overexpression of TLR4 and Na^+^/K^+^-ATPase, which regulates inflammation and cell volume. Furthermore, it was important to increase the uptake of glucose through metformin administration to diminish TLR4 and increase Na^+^/K^+^-ATPase expression (Rotter Sopasakis et al., 2019).

We hypothesized that the elevated Ca^2+^ signaling in OA chondrocytes would be normalized and return to physiological levels and that expression of the inflammatory mediator TLR4 would subsequently be diminished. Na^+^/K^+^-ATPase expression must increase for cell volume to normalize. By interfering with cellular events that are perturbed in OA chondrocytes, we propose that this drug combination can act as a DMOAD.

## 2 Methods and materials

### 2.1 Chondrocyte isolation and culture

Articular cartilage samples were obtained from euthanized horses within 24 ​h postmortem. Cartilage samples were collected from four horses and two different breeds (Standardbred trotter (n ​= ​2) and Swedish Warmblood horses (n ​= ​2). Both breeds can develop OA early in life because they start to train and race at an early age. The mean (±S.D) age of the horses was 4.75 ​± ​2.2. The aim was to collect cartilage from horses with early stages of OA; therefore, joints having macroscopic classification of mild structural OA relied on postmortem inspection of the radial facet of the middle carpal bone was collected. Articular cartilage samples from both left and right carpal joint was collected from one of the horses representing a healthy and an OA joint.

After slaughter, the left middle carpal joint was opened, and the articular cartilage of the proximal surface of the carpal bones was inspected macroscopically. The macroscopic classification of the joints was done by a pathologist at the university in Uppsala.

The extent of macroscopic cartilage lesions, as shown by superficial fraying and erosions, was smaller in the radial facet and classified as mild (Skiöldebrand et al., 2001).

The horses were euthanized for reasons unrelated to this study, and the Ethical Committee on Animal Experiments (Stockholm, Sweden) approved the study protocol (Dnr: N378/12).

Following aseptic preparation, cartilage of the dorsal aspect of the radial facet of the third carpal bone was incised with a scalpel, and full-thickness cartilage samples were collected with forceps. The tissue was placed in a sterile saline (0.9% NaCl) solution with gentamicin sulfate (50 ​mg/l) and amphotericin B (250 ​μg/ml). The cartilage samples were transported chilled (approx. 5 ​°C) to the laboratory. Isolation and expansion of chondrocytes were performed as previously described (Ley et al., 2011). The cells were expanded to passage 1 and then seeded at 20,000 ​cells/cm^2^ in chondrogenic medium to maintain the phenotype. The medium consisted of DMEM-glucose (5.5 ​mM) (Thermo Fisher Scientific; Waltham, MA, USA) supplemented with 14 ​μg/ml ascorbic acid (Sigma-Aldrich, St. Louis, MO, USA), 10^−7^ ​M dexamethasone (Sigma-Aldrich), 1 ​mg/ml human serum albumin (Equitech Bio, Kerville, TX, USA), 1 ​× ​insulin–transferrin–selenium (Gibco, Life Technologies, Carlsbad, CA, USA), 5 ​μg/ml linoleic acid (Sigma-Aldrich), 1 ​× ​penicillin-streptomycin (PEST) (Sigma-Aldrich), and 10 ​ng/ml human transforming growth factor (TGF) β-1 (R&D Systems, Abingdon, UK). The cells were grown to confluence for 5 days in 96-well culture plates for Ca^2+^ analysis or in 6-well culture plates for Western blot analysis. The cells were cultivated in 5.5 ​mM glucose (the physiological concentration) over the entire period.

### 2.2 Preincubation and drug treatment of OA chondrocytes

Chondrocytes (groups b, d, f and h) were preincubated with LPS (10 ​ng/ml) for 24 ​h on day 3 (Skiöldebrand et al., 2018; Block et al., 2013; Forshammar et al., 2011; Hansson et al., 2018).

On day 4, the confluent chondrocytes were incubated with LPS (groups b, d, f and h) or left unstimulated (groups a, c, e and g). Additional drugs were added to the media on day 4 and incubated with the chondrocytes for 24 ​h.

Groups:

a) No treatment (control)
b) LPS
c) Metformin ​+ ​bupivacaine
d) LPS ​+ ​metformin ​+ ​bupivacaine
e) Metformin ​+ ​sildenafil ​+ ​vitamin D3
f) LPS ​+ ​metformin ​+ ​sildenafil ​+ ​D3
g) Metformin ​+ ​bupivacaine ​+ ​sildenafil ​+ ​D3
h) LPS ​+ ​metformin ​+ ​bupivacaine ​+ ​sildenafil ​+ ​D3

LPS and metformin (1 ​mM) (Sigma Aldrich, bupivacaine (Marcain) (Astra Zeneca, Södertälje, Sweden) (10^−12^ ​M) (Block et al., 2013), sildenafil citrate salt (Sigma Aldrich) (1 ​μM) (S Nunes et al., 2016) and 1α,25-dihydroxyvitamin D3 (calcitriol) (Sigma-Aldrich) (100 ​nM) were used (Li et al., 2015).

### 2.3 Calcium imaging

On day 5, Ca^2+^ experiments were performed with the cells in 96-well plates, and the cells in 6-well plates were harvested for protein determination and Western blot analyses.

A high-throughput screening system was used to assess intra- and extracellular Ca^2+^ signaling (Flexstation 3 microplate reader, Molecular Devices, San José, USA). The cells were incubated with the Ca^2+^-sensitive probe FLIPR Calcium 6 (Molecular Devices) for 2 ​h. The cells were then exposed to different neurotransmitters: 5-HT (10^−5^ ​M), glutamate (10^−3^ ​M), or ATP (10^−4^ ​M), all from Sigma Aldrich (Saint Louis, USA), and Ca^2+^ release was immediately measured upon stimulation.

The total areas under the curve (AUCs) reflecting the amounts of Ca^2+^ released (Berridge, 2007) were analyzed to measure the Ca^2+^ responses. The amplitude (peak) is expressed as the maximum increase.

### 2.4 SDS-PAGE and western blotting

On day 5, the cells in 6-well plates were harvested for protein determination and Western blot analyses. The cells were rinsed twice in phosphate-buffered saline (PBS) and immediately lysed for 20 ​min on ice in cold radioimmunoprecipitation assay (RIPA) lysis buffer containing 150 ​mM NaCl, 1% IGEPAL® CA-630, 0.5% sodium deoxycholate, 0.1% SDS, and 50 ​mM Tris (pH 8.0) supplemented with a protease inhibitor cocktail containing 104 ​mM AEBSF, 80 ​μM aprotinin, 4 ​mM bestatin, 1.4 ​mM E−64, 2 ​mM leupeptin, and 1.5 ​mM pepstatin A. The procedure was performed according to the method of Persson et al. (2005). Separate aliquots were collected and used to determine the protein concentration. The samples were not denatured before loading onto the gels. All samples were analyzed for the total protein content, and 20 ​μg of the total protein from each sample was loaded in each lane of the gel. β-Actin was used as a control to ensure equal loading.

SDS-PAGE was conducted using the Novex precast gel system (Invitrogen) according to the manufacturer's recommendations with 4–12% Bis-Tris gels (Invitrogen) at 200 ​V for 50 ​min. The separated proteins were transferred at 30 ​V for 60 ​min to a nitrocellulose membrane (Invitrogen) using NuPAGE transfer buffer (Invitrogen) supplemented with methanol and NuPAGE antioxidant. The membranes were rinsed twice with distilled water, and the proteins were visualized with Ponceau S solution (Sigma Aldrich). The proteins were blocked with 0.5% fat-free skim milk (Semper AB, Götene, Sweden) in Tris-buffered saline (TBST; 50 ​mM Tris-HCl, 150 ​mM NaCl, and 0.05% Tween) for 60 ​min at room temperature. The membranes were probed with anti-TLR4 (rabbit polyclonal, 1:500) (SC 30002, Santa Cruz Biotech, Inc., Dallas, TX, USA), or a mouse monoclonal primary antibody against Na^+^/K^+^-ATPase (α-subunit) (A 276, Sigma Aldrich) diluted 1:250 and mouse monoclonal anti-β-actin (A 5441, Sigma Aldrich). Then, the samples were washed 4 ​× ​for 2 ​min with TBST, followed by incubation with secondary horseradish peroxidase (HRP)-conjugated antibodies [donkey anti-mouse or anti-rabbit F(ab’)_2_ fragment (Jackson ImmunoResearch) diluted 1:10000]. The samples were again washed several times in TBST. All of the primary and secondary antibodies were diluted in 0.5% fat-free skim milk in TBST. The antibody-bound protein was detected with an enhanced chemiluminescence kit (PerkinElmer, Inc., Waltham, MA, USA) and visualized using a Fujifilm LAS-3000 system (Tokyo, Japan).

### 2.5 Protein determination

A protein determination assay was performed in accordance with the manufacturer's instructions using a detergent-compatible (DC) protein assay (Bio-Rad, Hercules, CA, USA) based on the method used by Lowry and coworkers (Lowry et al., 1951) with minor modifications. The standard (0–4 ​mg/ml BSA) and samples were mixed with the reagents, and incubated for 15 ​min at room temperature, after which the absorbance at 750 ​nm was read with a VersaMax microplate reader and analyzed using SoftMax Pro 4.8 from Molecular Devices (Sunnyvale, CA, USA).

### 2.6 Statistics

Statistical analyses were performed with GraphPad 2.0 software (GraphPad, USA).

AUCs and peak values for Ca^2+^ signaling are presented as the mean and standard error of the mean (SEM). Differences in AUCs, peak measurements and integrated densities between the treatment groups were assessed using one-way ANOVA followed by Dunnett's multiple comparisons test. Significance was set at *P* ​< ​0.05.

## 3 Results

We previously showed that healthy chondrocytes show an extremely low level compared to OA chondrocytes (Skiöldebrand et al., 2018, 2019). The control cells in this study were inflamed OA chondrocytes.

### 3.1 Drug treatment and effects on calcium signaling

**Fig. 1:**
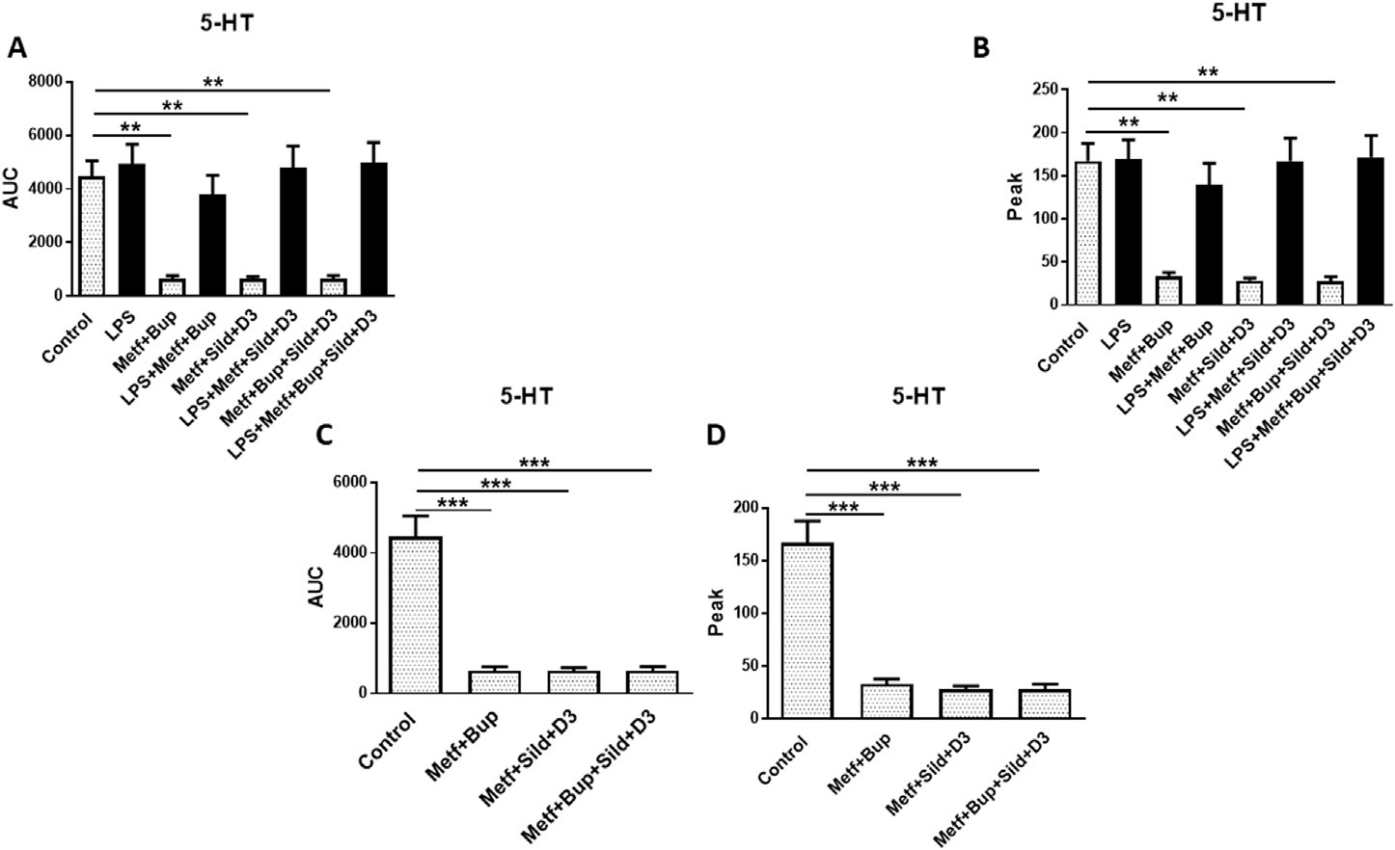
OA chondrocytes were stimulated in a fluorescence-based assay to detect changes in intracellular Ca^2+^ over time with 5-HT (10^−5^ ​M). The cells were cultivated in 5.5 ​mM glucose throughout the cultivation period. Ca^2+^ responses were measured after the cells were incubated with LPS (10 ​ng/ml) for 24 ​h, followed by LPS, metformin (1 ​mM), and bupivacaine (10^−12^ ​M); sildenafil (Sild) (1 ​μM) and vitamin D3 (D3) (100 ​nM); a combination of all substances for another 24 ​h; or the same as above except with LPS. Controls were untreated OA chondrocytes (A and B). The figures in C and D further clarify the results when chondrocytes were not treated with LPS. The area under the Ca^2+^ peak (AUC) was calculated for each Ca^2+^ transient (A and C), and the amplitude (peak) (B and D) is expressed as the maximum increase. The cells were assayed in 4 experiments in quadruplicate. The level of significance was analyzed using one-way ANOVA followed by Dunnett's multiple comparisons test. *∗∗P* ​< ​0.01, *∗∗∗P* ​< ​0.001.

Osteoarthritic chondrocytes that had been incubated with LPS for 24 ​h to further induce an inflammatory response were not further affected by LPS according to their Ca^2+^ signaling. Bupivacaine (1 pM) previously evaluated with a concentration curve (Block et al., 2013) in combination with metformin did not reduce the 5-HT-evoked intracellular Ca^2+^ release. Additional sildenafil (1 ​μM) in combination with vitamin D3 (100 ​nM) and metformin did not reduce 5-HT-evoked intracellular Ca^2+^ release. The combination of the four different pharmaceutical substances with metformin also did not reduce 5-HT-evoked intracellular Ca^2+^ release (Fig. 1 A, B).

Osteoarthritic chondrocytes that had not been incubated with LPS showed high 5-HT-evoked intracellular Ca^2+^ release (AUC 4307.7 ​± ​538.5), which was significantly diminished upon treatment with metformin and bupivacaine (615.4 ​± ​76.9, p ​< ​0.001); metformin, sildenafil and bupivacaine (615.4 ​± ​46.15, p ​< ​0.001); and metformin, sildenafil, bupivacaine and vitamin D3 (615.4 ​± ​61.5, p ​< ​0.001) (Fig. 1 A, C). Similar results were obtained for the 5-HT peak values (Fig. 1 B, D).

**Fig. 2:**
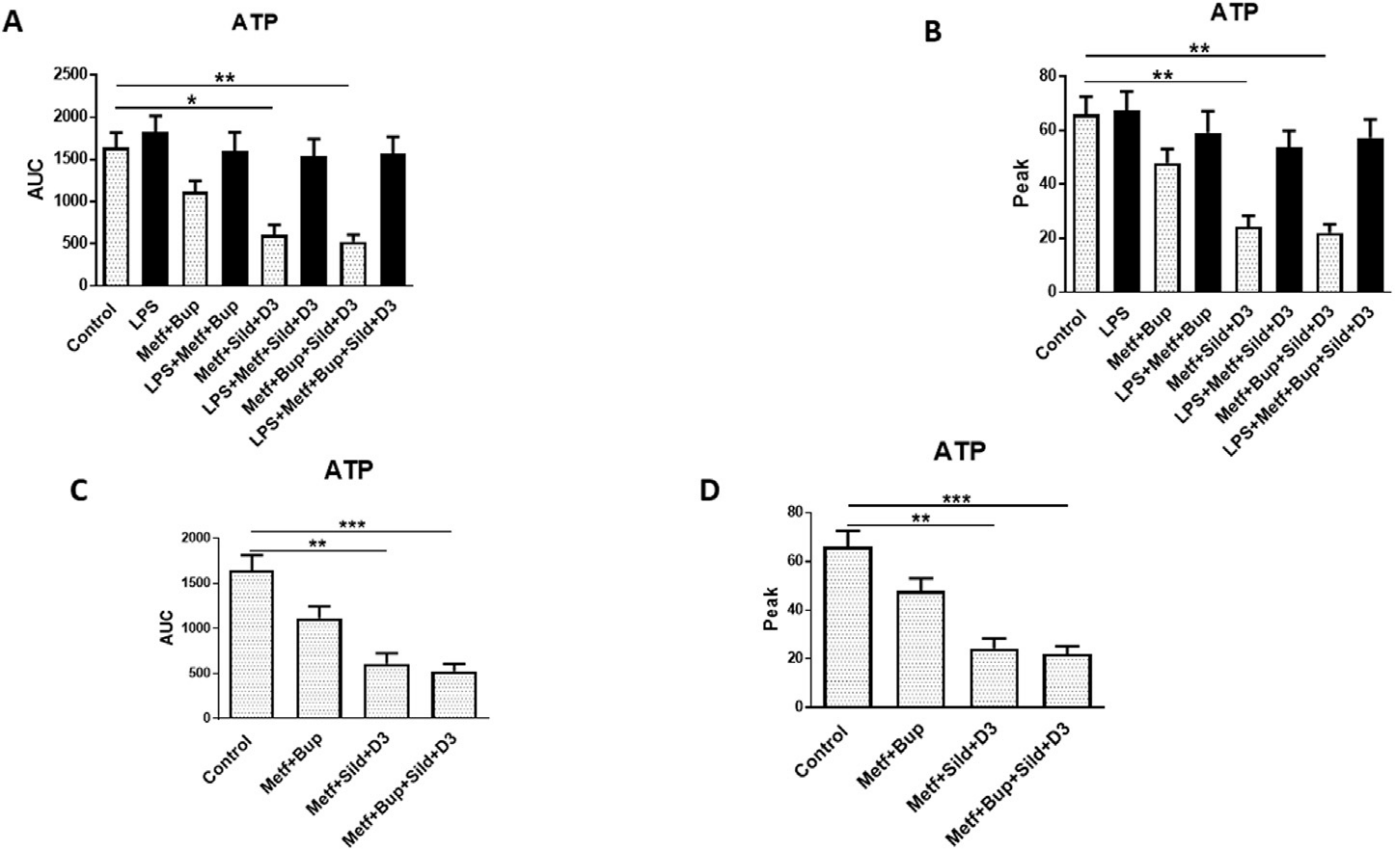
OA chondrocytes were stimulated in a fluorescence-based assay to detect changes in intracellular Ca^2+^ over time with ATP (10^−4^ ​M). The cells were cultivated in 5.5 ​mM glucose throughout the cultivation period. Ca^2+^ responses were measured after the cells were incubated with LPS (10 ​ng/ml) for 24 ​h, followed by LPS, metformin (1 ​mM), and bupivacaine (10^−12^ ​M); sildenafil (Sild) (1 ​μM) and vitamin D3 (D3) (100 ​nM); a combination of all substances for another 24 ​h; or the same as above except with LPS. Controls were untreated OA chondrocytes (A and B). The figures in C and D further clarify the results when chondrocytes were not treated with LPS. The area under the Ca^2+^ peak (AUC) was calculated for each Ca^2+^ transient (A and C), and the amplitude (peak) (B and D) is expressed as the maximum increase. The cells were assayed in 4 experiments in quadruplicate. The level of significance was analyzed using one-way ANOVA followed by Dunnett's multiple comparisons test. ∗*P* ​< ​0.05, *∗∗P* ​< ​0.01, *∗∗∗P* ​< ​0.001.

ATP-evoked intracellular Ca^2+^ release was affected by neither bupivacaine nor metformin in LPS-incubated cells. ATP-evoked intracellular Ca^2+^ release was also unaffected. The combination of these different pharmaceutical substances with metformin also did not reduce ATP-evoked intracellular Ca^2+^ release (Fig. 2 A, B).

Combination treatment with metformin, sildenafil, bupivacaine and vitamin D3 significantly reduced the AUC for ATP-evoked intracellular Ca^2+^ release by 525.0 ​± ​75.0 compared to that in untreated cells (1675.0 ​± ​175.0, p ​< ​0.01). The combination of metformin, sildenafil and vitamin D3 also significantly reduced the AUC for ATP-evoked intracellular Ca^2+^ release by 600.0 ​± ​115.0 compared to that in untreated cells (1675.0 ​± ​175.0, p ​< ​0.05) (Fig. 2A, C). Similar results were obtained for the ATP peak values (Fig. 2B, D).

**Fig. 3:**
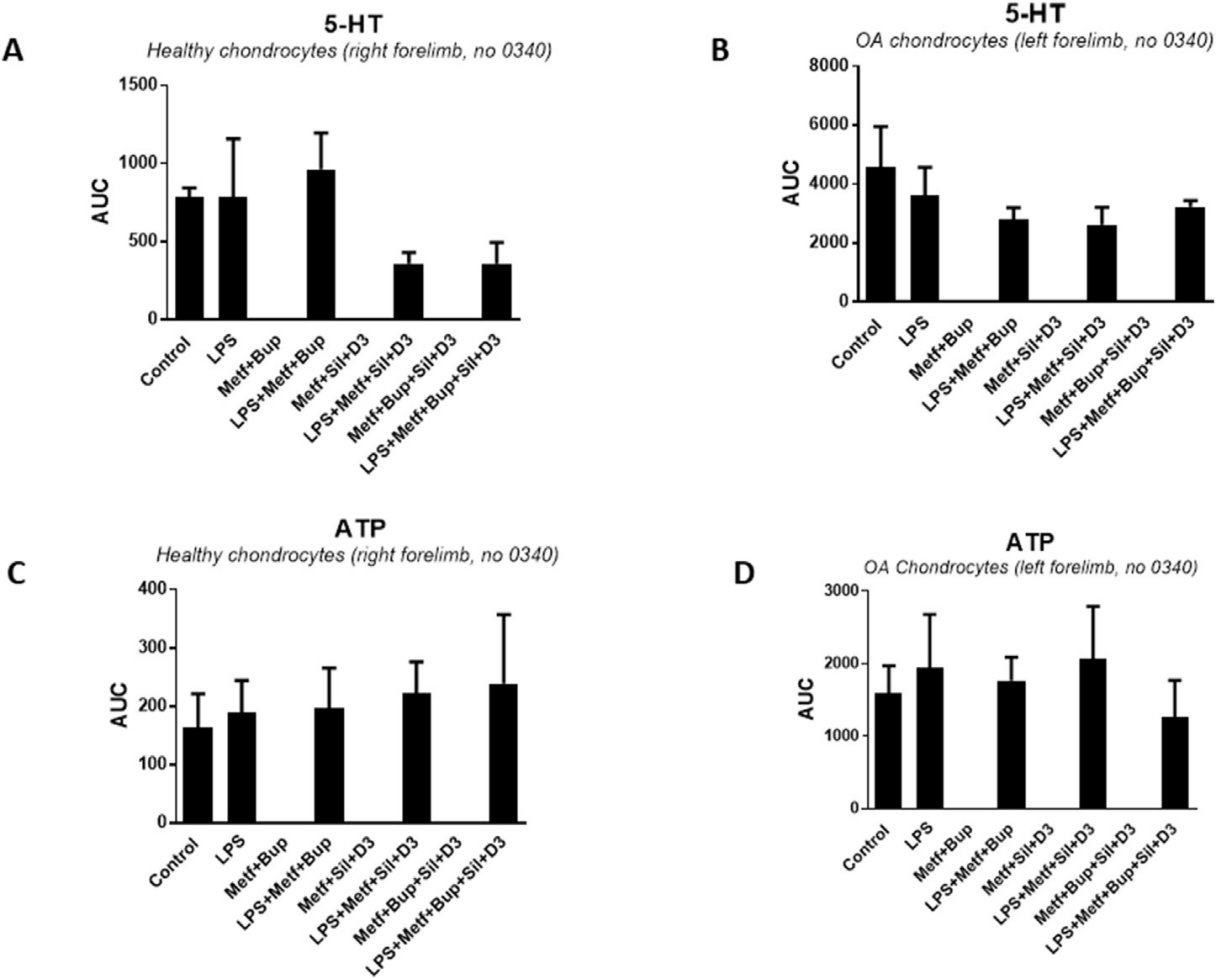
Healthy and OA chondrocytes from the same horse were stimulated in a fluorescence-based assay to detect changes in intracellular Ca^2+^ over time with 5-HT (10^−5^ ​M) or ATP (10^−4^ ​M). The cells were cultivated in 5.5 ​mM glucose throughout the cultivation period. Ca^2+^ responses were measured after the cells were incubated with LPS (10 ​ng/ml) for 24 ​h, followed by LPS, metformin (1 ​mM), and bupivacaine (10^−12^ ​M); sildenafil (Sild) (1 ​μM) and vitamin D3 (D3) (100 ​nM); a combination of all substances for another 24 ​h; or the same as above except with LPS. Controls were untreated healthy (A, C) and OA chondrocytes (B, D). The area under the Ca^2+^ peak (AUC) was calculated for each Ca^2+^ transient. The results for 5-HT for healthy chondrocytes (A) and OA chondrocytes (B). The results for ATP for healthy chondrocytes (C) and OA chondrocytes (D). The cells were assayed in one experiment.

Healthy and OA chondrocytes from the same horse showed a remarkable difference in their 5-HT evoked intracellular Ca^2+^ release. The healthy cells had a statistically significant lower AUC 785 ​± ​58 compared to OA chondrocytes AUC 4562 ​± ​1386 (n ​= ​5, p ​< ​0.05) (Fig. 3A and B).

No difference was obtained in ATP AUC for healthy and OA cells in the same horse (Fig. 3 C, D). Glutamate-evoked intracellular Ca^2+^ release was not affected in the osteoarthritic chondrocytes (data not shown).

### 3.2 Drug treatment and effects on inflammatory mediators

**Fig. 4:**
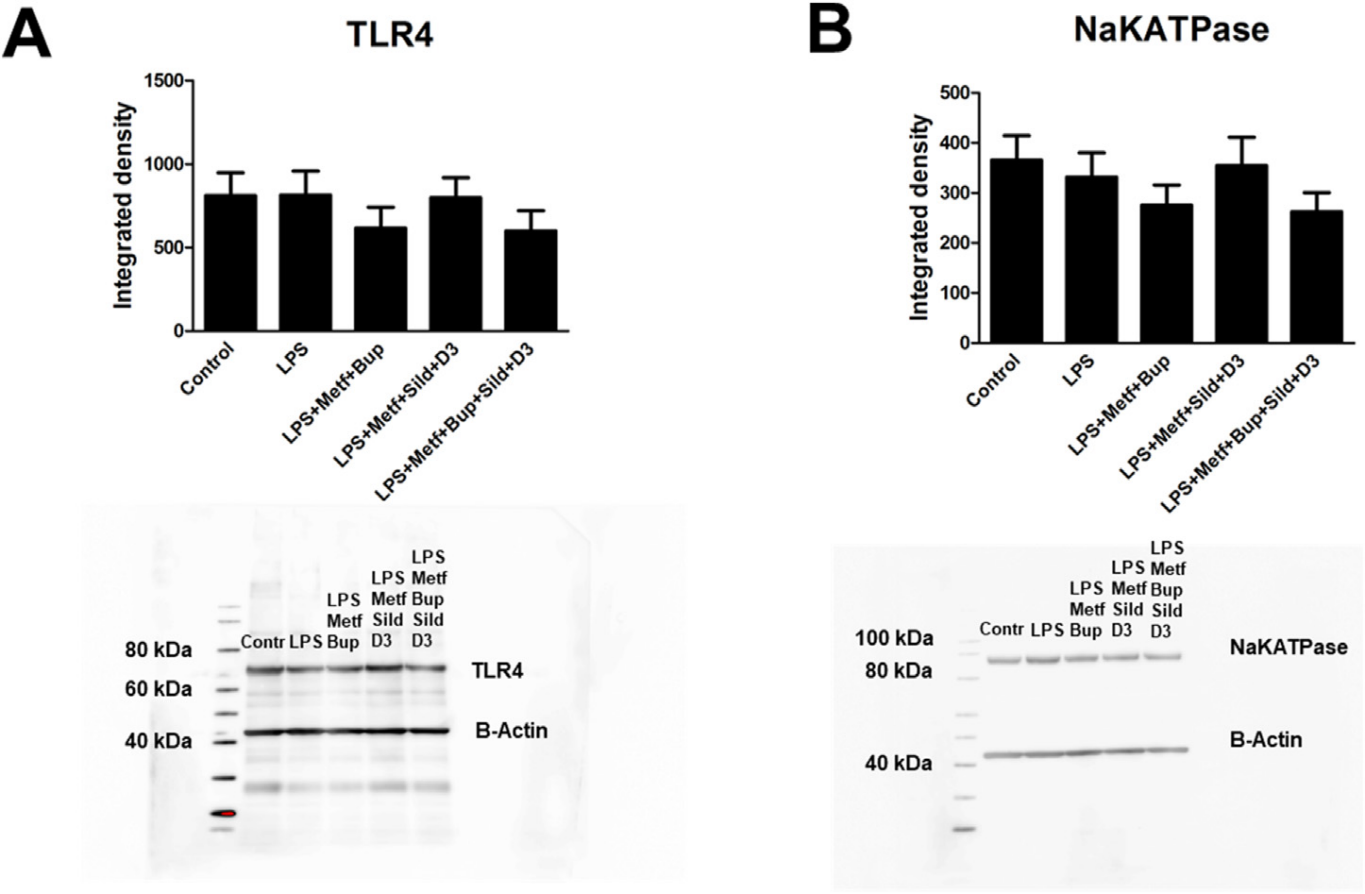
The expression levels of TLR4 (A) and Na^+^/K^+^-ATPase (B) were determined using Western blot analysis. OA chondrocytes were cultivated in 5.5 ​mM glucose for the entire cultivation period. The cells were incubated with LPS (10 ​ng/ml) for 24 ​h; LPS for 24 ​h followed by LPS, metformin (1 ​mM), bupivacaine (10^−12^ ​M); sildenafil (Sild) (1 ​μM) and vitamin D3 (D3) (100 ​nM) or a combination of all substances for an additional 24 ​h. Controls (Contr) were untreated OA chondrocytes. Significance was assessed using one-way ANOVA followed by Dunnett's multiple comparisons test. n ​= ​6. Representative images of Western blot membranes are presented.

**Fig. 5:**
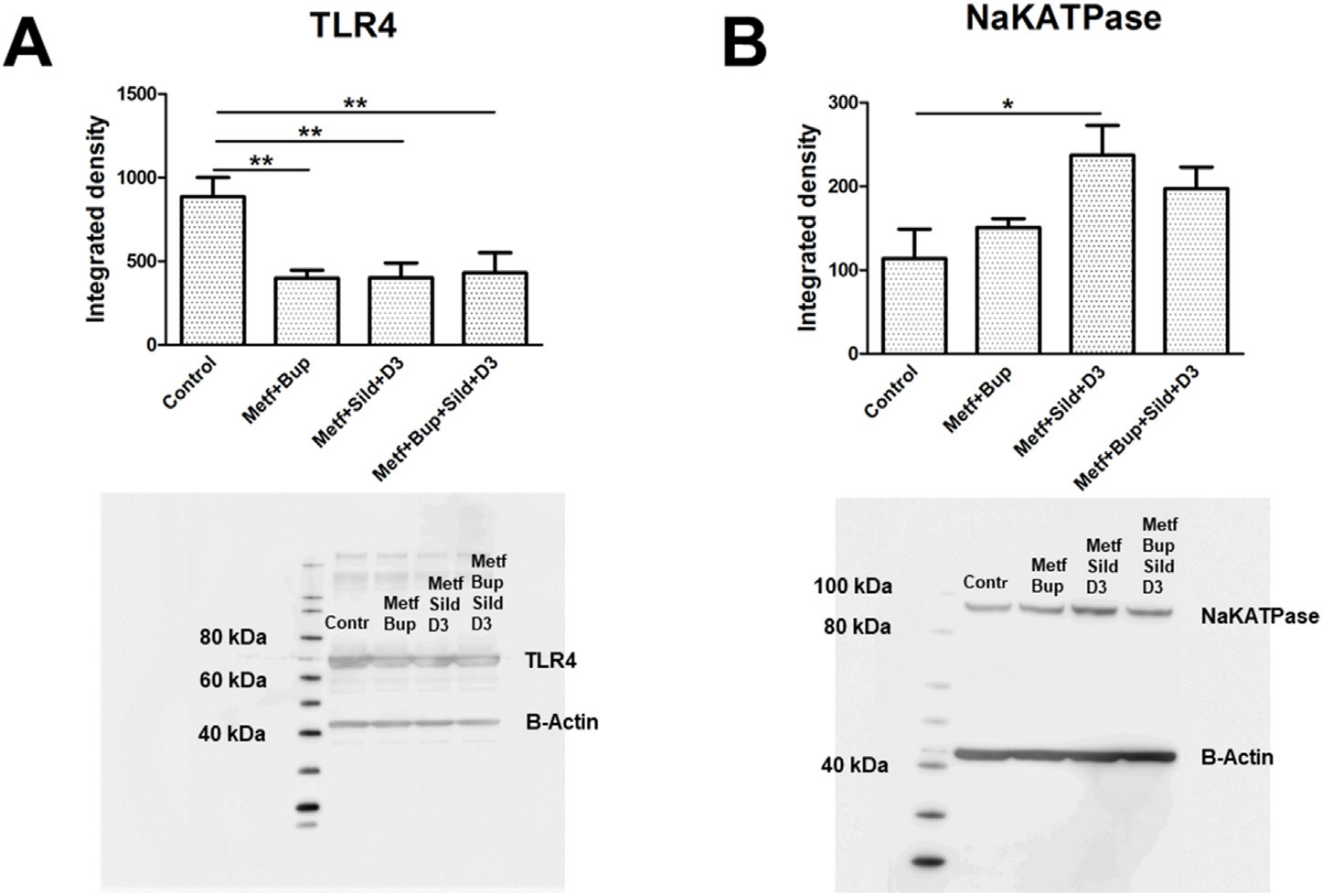
The expression levels of TLR4 (A) and Na^+^/K^+^-ATPase (B) were determined using Western blot analysis. OA chondrocytes were cultivated in 5.5 ​mM glucose for the entire cultivation period. The cells were incubated with metformin (1 ​mM), bupivacaine (10^−12^ ​M), sildenafil (Sild) (1 ​μM) and vitamin D3 (D3) (100 ​nM) or a combination of all substances for 24 ​h. Controls (Contr) were untreated OA chondrocytes. (D) Further clarification of the statistical results. Significance was assessed using one-way ANOVA followed by Dunnett's multiple comparisons test. *∗P* ​< ​0.05, *∗∗P* ​< ​0.01. n ​= ​6. Representative images of Western blot membranes are presented.

There was no change in the expression levels of TLR4 or Na^+^/K^+^-ATPase in LPS-incubated cells compared to cells without LPS incubation (Fig. 4 A, B). However, the chondrocytes without LPS incubation showed significantly decreased TLR4 expression after treatment with all drug combinations (Fig. 5 A). Combination treatment with metformin, sildenafil and vitamin D3 increased the expression of Na^+^/K^+^-ATPase (Fig. 5 B).

**Fig. 6:**
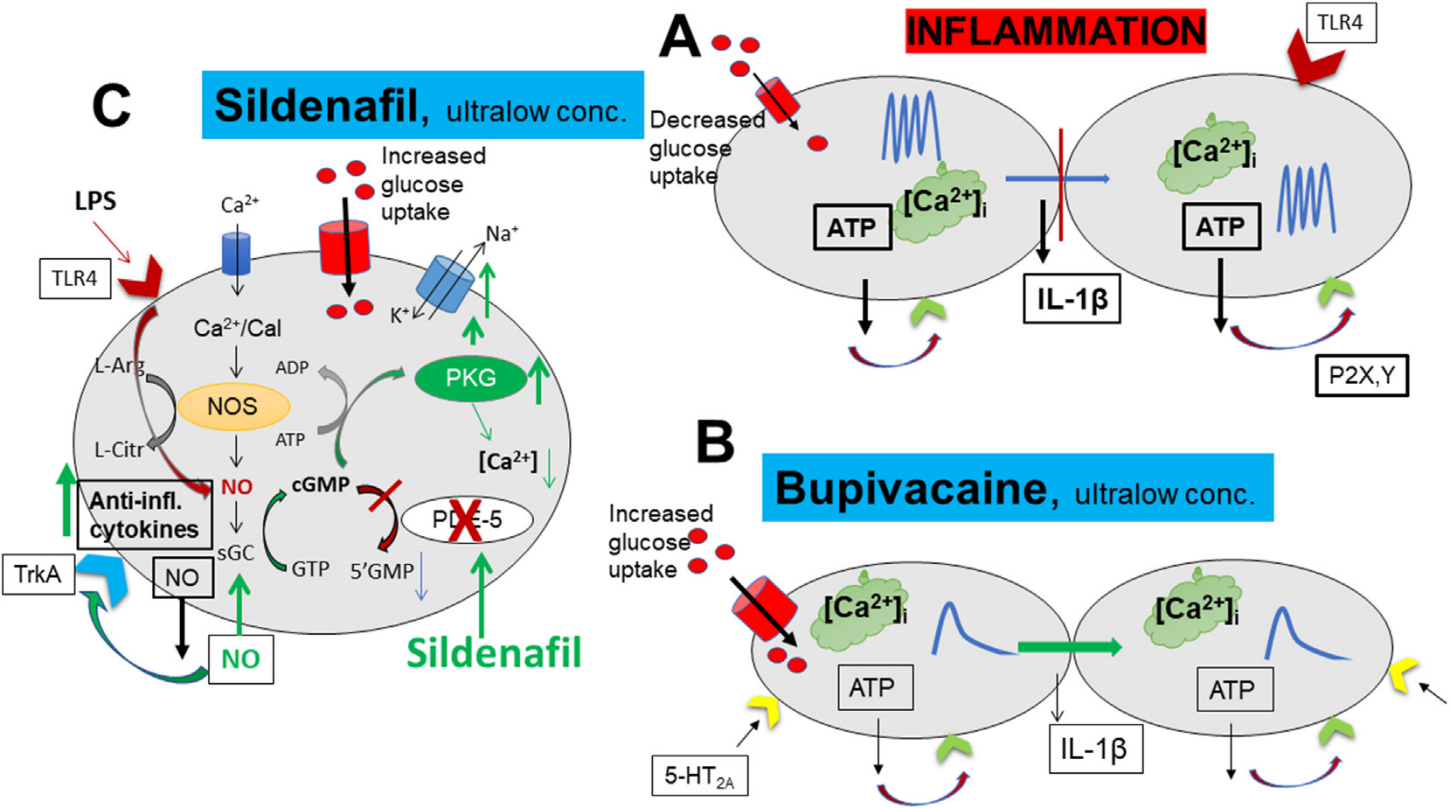
Schematic illustration highlighting inflammatory OA chondrocytes (A). Intracellular Ca^2+^ release is increased, resulting in Ca^2+^ oscillations. As a result, the production and release of ATP and proinflammatory cytokines increase. This in turn leads to extracellular Ca^2+^ signaling by the stimulation of ATP receptors. Intercellular Ca^2+^ signaling through gap junctions is decreased (Westhaus et al., 2017). Glucose uptake into chondrocytes is decreased (Rotter Sopasakis et al., 2019). (B) The local anesthetic agent bupivacaine (1 pM) reduces Ca^2+^ oscillations after stimulation with 5-HT. Intercellular Ca^2+^ signaling increases through gap junctions, and glucose uptake is increased. (C) Effects of sildenafil (1 ​μM), which blocks PDE-5 and increases the production of cGMP. This in turn increases protein kinase G (PKG) and stimulates the Na^+^/K^+^-ATPase pump. The expression of TLR4 is decreased. Even here the glucose uptake is increased. Through some mechanism, ATP production decreases (S Nunes et al., 2016).

An illustration of the results is shown in Fig. 6.

## 4 Discussion

Combination treatment with the drugs bupivacaine (1 pM) and sildenafil (1 ​μM), along with vitamin D3 (100 ​nM), was shown to restore elevated intracellular Ca^2+^ release and the expression of inflammatory factors in diseased chondrocytes. The reduced glucose uptake found in OA chondrocytes compared to control chondrocytes (Rotter Sopasakis et al., 2019) was restored by incubation of the cells with metformin along with the different drug combinations. This is in accordance with a previous study in astrocytes in which the combination of these drugs restored elevated 5-HT- and ATP-evoked intracellular Ca^2+^ signaling through gap junctions (Hansson and Skiöldebrand, 2019a). Bupivacaine (1 pM) was shown to attenuate 5-HT-evoked intracellular Ca^2+^ release in gap junction-coupled astrocytes (Block et al., 2013), and sildenafil (1 ​μM) also attenuated ATP-evoked intracellular Ca^2+^ release in these cells (S Nunes et al., 2016). The extremely low concentrations of bupivacaine and sildenafil are much lower than usually used to avoid presumably negative well-known effects. These concentrations seem to have anti-inflammatory effects.

The prominent downregulation of TLR4 in chondrocytes treated with metformin and bupivacaine further indicates that this drug combination has anti-inflammatory properties.

TLR4 expression was shown to be upregulated under inflammatory conditions and in patients with OA (Hutchinson et al., 2008; Gómez et al., 2015). Activation of TLR4 leads to the activation of nuclear factor κB (NFқB), a factor that regulates the expression of genes involved in events in the immune response, including the release of proinflammatory cytokines such as TNF-α and IL-1β and induction of MMPs (Kielian, 2006; Gómez et al., 2015). TLR4-caused inflammation in OA was shown to induce cartilage degradation and decrease collagen type II and aggrecan synthesis, and different blockers that modulate TLR4 signaling in joint tissues have been proposed as DMOADs (Gómez et al., 2015).

Substantial progress has been made in understanding how chronic systemic low-grade inflammation influences the physiology of several diseases, but why this inflammation fails to resolve in OA, metabolic syndromes, and autoimmune diseases is unclear. The physiological characteristics of inflammation are key contributors to these conditions, and an increasing number of studies investigating inflammatory markers have been carried out. Immune cells are involved in defense mechanisms against pathogens (Perretti et al., 2015). Inflammation requires contributions from other tissue cells, such as supportive cells in the body, which are connected by gap junctions to form large networks. Such supportive cells depend on the affected organ and include astrocytes, keratinocytes, chondrocytes, synovial fibroblasts, osteoblasts, connective tissue cells, cardiac and corneal fibroblasts, myofibroblasts, hepatocytes, and different types of glandular cells (Hansson and Skiöldebrand, 2015, 2019b). Several organs make up these systems, and these cells are excitable but do not display action potentials. Furthermore, these systems are equipped with intercellular and/or extracellular Ca^2+^ signaling systems (Skiöldebrand et al., 2018; D'Andrea and Vittur, 1996; Matta and Zakany, 2013; Carpintero-Fernandez et al., 2018).

We previously showed that healthy and osteoarthritic chondrocytes exhibited different inflammatory responses and reacted differently to 5-HT-evoked Ca^2+^ signaling, glutamate concentrations in culture media, F-actin content, and the expression of μ- and κ-opioid receptors (Skiöldebrand et al., 2019). We have also shown that chondrocytes from healthy horses exposed to LPS for 24 ​h reached an inflammatory state with slightly increased intracellular Ca^2+^ release and TLR4 expression (Skiöldebrand et al., 2018). LPS is a potent inflammatory activator (Wang et al., 2014) that stimulates TLR4 (Kielian, 2006). However, compared to chondrocytes from healthy horses, OA chondrocytes (without LPS exposure) showed even more excessive Ca^2+^ signaling, a further enhanced inflammatory status and increased activity of the factors we analyzed (Skiöldebrand et al., 2019). Therefore, in this study, untreated OA chondrocytes (without LPS incubation) were chosen to serve as a control for comparison with treated cells from the same horse. This is in our opinion the most relevant type of cells for such a comparison, as our objective was to decrease inflammatory factors by treating inflamed OA chondrocytes with pharmaceutical substances. In the present study, we exposed half of the OA chondrocytes to LPS for 24 ​h to further increase their inflammatory activity. Unexpectedly, these chondrocytes did not show further inflammatory activation. Moreover, neither 5-HT- or ATP-evoked intracellular Ca^2+^ release nor expression of the inflammatory receptor TLR4 nor Na^+^/K^+^-ATPase activity in the cells could be restored with different combinations of the pharmaceutical substances. We hypothesized that these cells were already in a highly inflammatory state; thus, further activation was not possible. In this state, changes in the cells could not be restored, and the cells were unaffected by the indicated treatments. We became thoughtful and surprised that OA chondrocytes did not respond to LPS. Previous results by Li et al. (2020) showed that LPS could not promote inflammation in alveolar epithelial cells but in macrophages cells, whereas an amplified inflammatory response was observed in co-cultured of the two cells. Different concentrations and incubation times of LPS was also explained for the unchanged inflammatory response of the celltypes (Li et al., 2020). One may speculate that co-culture of synoviocyte with chondrocyte tense another response to LPS as the cross talk between the cells are necessary for the progression of OA (Chou et al., 2020).

We previously found that chondrocytes with inflammatory activation consume more glucose than healthy chondrocytes and that glucose transporter proteins (GLUTs) are downregulated and the glucose balance is disrupted in OA chondrocytes (Rotter Sopasakis et al., 2019; Mobasheri et al., 2002). Chondrocytes consume glucose as a primary substrate for ATP production in glycolysis, and these cells express several GLUT isoforms. Glucose is also an important substrate for matrix molecule synthesis, as high-molecular-weight hyaluronan is important for the assembly of articular cartilage (Rotter Sopasakis et al., 2019). Exposure of cells to metformin affects cell metabolism by increasing cellular glucose consumption and thereby enhancing glycolytic flux through the cell membrane (Westhaus et al., 2017). These results have been observed in muscle cells, and an anti-inflammatory effect was found to be mediated by inhibition of NFқB (Saisho, 2015).

The local anesthetic agent bupivacaine at extremely low concentrations (1 pM) was shown to have anti-inflammatory effects on 5-HT-evoked intracellular Ca^2+^ release in gap junction-coupled astrocyte networks (Block et al., 2013). Bupivacaine in combination with metformin restored 5-HT-evoked intracellular Ca^2+^ release and attenuated the expression of TLR4. We also assessed the effects of the substances alone in single experiments but did not obtain significant results; these data are not shown in this study.

ATP is involved in all cellular signaling systems, and increased ATP release from excitable cells leads to increased extracellular Ca^2+^ signaling (Cotrina et al., 2000; Vardjan and Zorec, 2015). An enhanced extracellular ATP concentration has been observed in inflammatory diseases (Beamer et al., 2017). We also observed increased ATP-evoked Ca^2+^ signaling in inflammatory-induced chondrocytes (Skiöldebrand et al., 2018) as well as inflammatory-induced astrocytes (Hansson and Skiöldebrand, 2019a), and we hypothesized that ATP-evoked Ca^2+^ signaling could be decreased to the physiological level.

The potent and selective PDE-5 inhibitor sildenafil (Peixoto et al., 2015) has been shown to have anti-inflammatory and neuroprotective effects, possibly through modulation of AMP-activated protein kinase (AMPK) and NFқB signaling (Peixoto et al., 2015). AMPK also promotes glucose uptake (O'Neill, 2013). Furthermore, sildenafil reduces expression of the proinflammatory cytokines IL-1β and TNF-α (Nunes et al., 2015). These results suggest that the cGMP pathways may regulate responses after inflammatory induction, including changes to the cellular cytoskeleton (Borán and Garcia, 2007).

Vitamin D3, or 1α,25-dihydroxyvitamin D3, a neuroprotective hormone that activates its receptor, VDR, protects against blood-brain barrier (BBB) disruption in endothelial cells in microvessels (Enkhjargal et al., 2017). VDR is expressed in all tissues, including chondrocytes (Haussler et al., 2013). Vitamin D3 acts as an immune regulator and a stimulator of neurotrophic factors and neurotransmitter expression (Jo et al., 2015). Chondrocytes can produce vitamin D metabolites under the regulation of growth factors and hormones, and these metabolites play an important role in the regulation of matrix vesicles (Assmusen et al., 2017). Vitamin D3 has been suggested to dampen OA, which may indicate a relationship between vitamin D3 and OA (Hansen et al., 2017). We observed that vitamin D3 downregulates TLR4 and decreases TNF-α and IL-6 release (Jo et al., 2015; Adamczak, 2015). As vitamin D3 attenuates inflammatory responses, we included it in our drugs to restore inflammatory chondrocytes.

Many low-grade chronic inflammatory diseases have been studied in research animals (both in vitro and in vivo) that do not spontaneously develop the disease. To the best of our knowledge, the early disease events of OA can be characterized and studied in chondrocytes from OA horses, which are prone to develop OA early in life due to the strenuous load that they are subjected to join during high-speed training and competition.

## 5 Conclusion

We found that a drug combination consisting of bupivacaine (1 pM) and sildenafil (1 ​μM), both at extreme low concentrations not usually used, in addition to vitamin D3 had unexpected beneficial effects on cellular systems that are perturbed in OA chondrocytes. The effects of this drug combination on 5-HT- and ATP-evoked intracellular Ca^2+^ release as well as expression of the inflammatory receptor TLR4 was reduced in the presence of metformin and bupivacaine at very low concentrations. Sildenafil and vitamin D3 increased Na^+^/K^+^-ATPase expression, and the combination of all three substances reduced TLR4 expression. These anti-inflammatory effects were enhanced by metformin. These findings suggest that this drug combination has the properties of a DMOAD. Further studies on synthesis and degradation of the ECM in 3D-cultured OA chondrocytes and thereafter, clinical studies first in horses with OA and then in humans with OA.

## CRediT authorship contribution statement

**Elisabeth Hansson:** Conceptualization, Data curation, Formal analysis, Funding acquisition, Investigation, Methodology, Project administration, Resources, Software, Supervision, Validation, Visualization, Writing – original draft, Writing – review & editing. **Eva Skiöldebrand:** Conceptualization, Data curation, Formal analysis, Funding acquisition, Investigation, Methodology, Project administration, Resources, Software, Supervision, Validation, Visualization, Writing – original draft, Writing – review & editing.

## Declaration of competing interest

The authors declare no potential conflicts of interest with respect to the research, authorship, or publication of this article.

## Abbreviations

PDE-5: phosphodiesterase-5
vitamin D3: 1α,25-dihydroxyvitamin D3
TLR4: Toll-like receptor 4
TNF-α: tumor necrosis factor-α
IL-1β: interleukin-1β
LPS: lipopolysaccharide
VDR: vitamin D receptor
AMPK: AMP-activated protein kinase
PBS: phosphate-buffered saline
RIPA: radioimmunoprecipitation assay
GLUT: glucose transporter
TGF β-1: transforming growth factor β-1
BSA: bovine serum albumin
AUC: area under the curve
OA: osteoarthritis
BBB: blood-brain barrier

## Language editing

We thank Springer Nature Author Services for language editing.
